# Harnessing Diesel-Degrading Potential of an Antarctic Microalga from Greenwich Island and Its Physiological Adaptation

**DOI:** 10.3390/biology12081142

**Published:** 2023-08-17

**Authors:** Zheng Syuen Lim, Chiew-Yen Wong, Siti Aqlima Ahmad, Nurul Aini Puasa, Lai Yee Phang, Noor Azmi Shaharuddin, Faradina Merican, Peter Convey, Azham Zulkharnain, Hasrizal Shaari, Alyza Azzura Azmi, Yih-Yih Kok, Claudio Gomez-Fuentes

**Affiliations:** 1 School of Health Sciences, International Medical University, Bukit Jalil, Kuala Lumpur 57000, Malaysia; syuenylim@gmail.com (Z.S.L.); yihyih_kok@imu.edu.my (Y.-Y.K.); 2 Department of Biochemistry, Faculty of Biotechnology and Biomolecular Sciences, Universiti Putra Malaysia, Serdang 43400, Selangor, Malaysia; nurulainipuasa@gmail.com (N.A.P.); noorazmi@upm.edu.my (N.A.S.); 3 Centre for Environmental and Population Health, Institute for Research, Development and Innovation (IRDI), International Medical University, Bukit Jalil, Kuala Lumpur 57000, Malaysia; 4 Center for Research and Antarctic Environmental Monitoring (CIMAA), Universidad de Magallanes, Avda. Bulnes, Punta Arenas 01855, Chile; claudio.gomez@umag.cl; 5 Laboratory of Bioresource Management, Institute of Tropical Forestry and Forest Products (INTROP), Universiti Putra Malaysia, Serdang 43400, Selangor, Malaysia; 6 Material Synthesis and Characterization Laboratory, Institute of Advanced Technology, Universiti Putra Malaysia, Serdang 43400, Selangor, Malaysia; 7 Department of Bioprocess Technology, Faculty of Biotechnology and Biomolecular Sciences, Universiti Putra Malaysia, Serdang 43400, Selangor, Malaysia; phanglaiyee@upm.edu.my; 8 School of Biological Sciences, Universiti Sains Malaysia, Minden 11800, Pulau Pinang, Malaysia; faradina@usm.my; 9 British Antarctic Survey, NERC, High Cross, Madingley Road, Cambridge CB3 0ET, UK; pcon@bas.ac.uk; 10 Department of Zoology, University of Johannesburg, P.O. Box 524, Auckland Park 2006, South Africa; 11 Millennium Institute Biodiversity of Antarctic and Subantarctic Ecosystems (BASE), Las Palmeras 3425, Ñuñoa, Santiago 7750000, Chile; 12 Department of Bioscience and Engineering, College of Systems Engineering and Science, Shibaura Institute of Technology, 307 Fukasaku, Minuma-ku, Saitama 337-8570, Japan; azham@shibaura-it.ac.jp; 13 Centre of Research and Field Services, Universiti Malaysia Terengganu, Kuala Nerus 21030, Terengganu, Malaysia; riz@umt.edu.my; 14 Institute of Oceanography and Environment, Universiti Malaysia Terengganu, Kuala Nerus 21030, Terengganu, Malaysia; 15 Faculty of Science and Marine Environment, Universiti Malaysia Terengganu, Kuala Nerus 21030, Terengganu, Malaysia; alyza.azzura@umt.edu.my

**Keywords:** Antarctic microalga, phytoremediation, diesel biodegradation, biosorption, hydrocarbon

## Abstract

**Simple Summary:**

Phytoremediation is a plant-based approach to extract, stabilise, eliminate, or render pollutants into less harmful form. The study highlights the use of a native polar microalga as a means of phytoremediation in Antarctica where imported microbes are prohibited. Since 1959, Antarctica has been a protected region to preserve its dynamic ecosystems, but it is increasingly vulnerable to climate change and pollution. One of the anthropogenic disturbances in the continent is diesel spillage. Due to the extreme polar environment, natural attenuation of spilled diesel is severely hindered; hence, the problem calls for an effective and sustainable solution. This laboratory study proved that Antarctic microalga was capable of removing diesel (57.6%) through biodegradation and biosorption in the span of nine days. Meanwhile, mixotrophic cultivation triggered the vacuolar activities and potentially stimulated lipid assimilation in the cells. The microalgal-based process offers a cheap alternative in water decontamination while bearing the economic potential through the secretion of valuable products, such as biolipids.

**Abstract:**

Microalgae are well known for their metal sorption capacities, but their potential in the remediation of hydrophobic organic compounds has received little attention in polar regions. We evaluated in the laboratory the ability of an Antarctic microalga to remediate diesel hydrocarbons and also investigated physiological changes consequent upon diesel exposure. Using a polyphasic taxonomic approach, the microalgal isolate, WCY_AQ5_1, originally sampled from Greenwich Island (South Shetland Islands, maritime Antarctica) was identified as *Tritostichococcus* sp. (OQ225631), a recently erected lineage within the redefined *Stichococcus* clade. Over a nine-day experimental incubation, 57.6% of diesel (~3.47 g/L) was removed via biosorption and biodegradation, demonstrating the strain’s potential for phytoremediation. Fourier transform infrared spectroscopy confirmed the adsorption of oil in accordance with its hydrophobic characteristics. Overall, degradation predominated over sorption of diesel. Chromatographic analysis confirmed that the strain efficiently metabolised medium-chain length *n*-alkanes (C-7 to C-21), particularly *n*-heneicosane. Mixotrophic cultivation using diesel as the organic carbon source under a constant light regime altered the car/chl-a ratio and triggered vacuolar activities. A small number of intracellular lipid droplets were observed on the seventh day of cultivation in transmission electron microscopic imaging. This is the first confirmation of diesel remediation ability in an Antarctic green microalga.

## 1. Introduction

Pelagic phytoplankton along with sea-ice and benthic algae are major primary producers in Antarctic marine ecosystems. Microalgae thrive in some of the harshest environments on Earth and contribute 55–65% of total primary production in the polar regions [1,2]. In recent years, microalgae have increasingly been exploited in the biopharmaceutical, renewable energy, nutraceutical, and wastewater industries, amongst others. They have several key advantages in industrial applications, including high surface-area-to-volume ratio, rapid metabolism, wide distribution, low cost, and limited and economically viable nutrient requirements [3]. However, to date most research in the field of bioremediation has focused on tropical microalgae with only limited attention given to polar species.

A recent bibliometric analysis of reports of hydrocarbon pollution in Antarctica during the period of 1980–2020 highlighted the occurrence of diesel release in shipping incidents and from pipelines and other facilities around research stations [4]. The most catastrophic incidents of all time that happened in the Antarctic water is the grounding of Bahía Paraíso in Arthur Harbour in 1989, causing an estimated spillage of 600,000 L of diesel fuel arctic (DFA) around Palmer Station [5].One recent example is the accidental release of 4000 L of Special Antarctic Blend (SAB) diesel fuel at Casey Station in 2015 [6]. The rate of breakdown of hydrophobic organic compounds in Antarctica is limited by the chronically cold climate, while logistic practicalities limit access to clean-up infrastructure. Diesel toxicity towards different organisms is a concerning aftermath due to the persistence of petrogenic contaminants in the cold region [7,8]. In light of that, effective management of diesel-polluted sites requires the use of efficient and economic technologies to contain and mitigate the contamination. In some parts of the world, hydrocarbon-degrading bacterial strains are used as a means of diesel biodegradation [9,10]; however, the decreased oxygen saturation levels that develop during hydrocarbon metabolism has led to the strategy being questioned [11]. Algae provide a potential alternative and sustainable strategy to overcome such drawbacks.

Phytoremediation refers to the use of macro- and microalgae as remediating agents. A range of microalgal taxa has been reported with specific bioremediation abilities, including members of the genera *Chlorella*, *Scenedesmus*, *Selenastrum*, *Phormidium*, *Botryococcus*, *Chlamydomonas*, *Spirulina*, *Oscillatoria*, *Desmodesmus,* and *Arthrospira*, generally active against single hydrocarbon compounds, such as phenanthrene, naphthalene, and pyrene [12]. The possible metabolic pathways are usually aerobic processes involving cytochrome P450 monooxygenase, dioxygenase, and glutathione-S-transferase [13,14,15]. Few studies have explored the application of microalgae in the remediation of the complex hydrocarbon mixtures that are generally typical in pollution events, for instance those involving diesel and crude oil. Algal behaviour and degradation performance may vary due to the multitude of possible interactions between them and the various hydrocarbons in the mixture [16,17]. It is also reported that microalgae demonstrated preferential hydrocarbons of varying lengths and compound structures [16,18].

Antarctic algae are less sensitive to and less impacted by diesel pollutants compared to marine invertebrates [19]. Psychrophilic algae can alter their physiology in response to varying environmental conditions, in particular by upregulating the production of proteins and ribosomes, accumulating compatible solutes, increasing membrane fluidity, and producing exopolymers and ice binding proteins [20]. This high adaptability enhances their survival.

This study set out to assess the potential of an Antarctic microalga in the remediation of diesel hydrocarbons. Many representatives of green algae are potential candidates for phytoremediation. Amongst these, the subaerial green algae have not been widely explored, a group which displays high phenotypic plasticity. We applied a polyphasic approach to identify the studied microalga based on morphological and molecular approaches. Furthermore, as information on algal physiological responses to diesel is lacking, we also examined physiological changes at the cellular level associated with exposure to diesel using transmission electron microscopy (TEM).

## 2. Materials and Methods

### 2.1. Microalgal Culture

The studied microalgal strain, WCY_AQ5_1, was preliminarily screened to identify its diesel-degrading potential prior to the study. The alga was originally isolated by the corresponding author in 2016 from Greenwich Island (South Shetland Islands, maritime Antarctica). The monoculture was grown in liquid Bold’s basal medium (BBM) [21], incubated at 4 °C, and illuminated with cool white fluorescent lamps (Philips, TLD 18W/54-765 providing 42 µmol m^−2^ s^−1^ PAR) on a 12:12-h light–dark cycle. The culture was aerated by a mechanical air pump (containing a 0.22 µm filter) and maintained in the exponential growth phase through repeated sub-culturing with fresh medium every week.

### 2.2. Growth Characterisation

Dry biomass of algal cells was measured after filtration through Whatman glass microfiber filters GF/C^TM^. Algal growth was measured daily by assessing chlorophyll *a* (chl-*a*) content, which was determined by spectrophotometry after pigment extraction in methanol [22]. The concentrations of chl-*a*, chlorophyll *b* (chl-*b*), and total carotenoids (car) were calculated using the following equations:

(1) $$chl\;a\left(mgL^{-1}\right)={16.72A}_{665.2}-{9.16A}_{652.4}$$

(2) $$chl\;b\left(mgL^{-1}\right)={34.09A}_{652.4}-{15.28A}_{665.2}$$

(3) $$car\left(mgL^{-1}\right)=\frac{1000A_{470}-1.63_{ca}-104.96_{cb}}{221}$$

where *A* is the absorbance at the subscript specified wavelength and *ca* and *cb* are the concentrations of chl-*a* and chl-*b*, respectively. The specific growth rate (*µ*) was determined using the formula [23]:

(4) $$\mu =\frac{{lnN}_{t1}-{lnN}_{t0}}{t_{1}-t_{0}}$$

where, *N_t_*_0_ and *N_t_*_1_ represent chl-*a* at *t*_0_ and *t*_1_, respectively, within the exponential phase.

### 2.3. Polyphasic Strain Identification and Characterisation of Surface Properties

#### 2.3.1. Morphological Identification

Morphological features were observed using light microscopy (Olympus BX53, Tokyo, Japan), phase contrast microscopy, scanning electron microscopy (SEM) (JEOL JSM-6400, Tokyo, Japan), and transmission electron microscopy (TEM) (JOEL JEM-2100F, Tokyo, Japan). Diacritical characteristics, including cell size and shape, arrangement, and presence of pyrenoid, were recorded and compared to the descriptions in the reference key of John, Brooks, and Whitton [24] and Pröschold and Darienko [25]. Size measurements were made on 30 randomly selected cells under light microscopy. For SEM analysis, the cells were fixed with 4% glutaraldehyde, washed with 0.1 M sodium cacodylate buffer, post-fixed with 1% OsO_4_, and dehydrated in an acetone series (35–100%) sequentially. Specimens were critical-point dried and gold sputtered before being viewed at varying magnification ranges using the SEM.

For TEM, samples were embedded into beam capsules, which were then filled with resin. Thick sections of 1 µm were cut using an ultramicrotome, followed by ultrathin sectioning of the area of interest. Observations were carried out using a field emission TEM.

#### 2.3.2. Molecular Identification Using a Dual Barcode Approach

Genomic DNA was extracted from the microalgal culture using the NucleoSpin DNA Plant Kit (Machnerey-Nagel, Düren, Germany), following the manufacturer’s instructions. The 18S rDNA gene was PCR-amplified using the universal eukaryotic primers, 20F (5′-GTA GTC ATA TGC TTG TCT C-3′) and 18 L (5′-CAC CTA CGG AAA CCT TGT TAC GAC TT-3′) [26]. Thermal cycling conditions were set at 95 °C for 5 min for pre-denaturation, followed by 35 cycles of denaturation at 94 °C for 1 min, 56 °C for 1 min, and 72 °C for 3 min, with a final extension at 72 °C for 10 min. The internal transcribed spacer (ITS) region was PCR-amplified using the primers, ITS_1 (5′-TCC GTA GGT GAA CCT GCG G-3′) and ITS_4 (5′-TCC TCC GCT TAT TGA TAT GC-3′). The thermal cycling conditions used were 94 °C for 2 min for pre-denaturation, followed by 35 cycles of denaturation at 94 °C for 30 sec, 54 °C for 30 sec, and 72 °C for 30 sec, with a final extension at 72 °C for 7 min. For both amplifications, 25 μL PCR mixture was prepared as follows: template DNA 2 μL, 12.5 μL 2X ViRed Taq Master Mix (Vivantis), 1 μL of each primer in final concentration, and 8.5 μL dH_2_O. The integrity of the PCR products was verified using a 1% agarose gel prepared in 1× Trisacetate-EDTA (TAE) buffer before sending for Sanger sequencing (BioBasic, Singapore). The expected amplicon sizes were 1700 bp for the 18S rDNA and 600 bp for the ITS region.

The sequences obtained were edited, aligned, and concatenated using Geneious 11.0 (Biomatters, http://www.geneious.com, 19 November 2022) and then searched against the GenBank database using the basic local alignment search tool (BLAST). Sequences with cut-off values of 97% and above were selected, and uncultured strains were excluded. Sequence alignment was performed for the isolated strain and reference sequences from GenBank using the MUSCLE algorithm. Phylogenetic analysis was conducted using the software package MEGA version X based on the maximum likelihood method (ML) and Kimura 2-parameter model with 1000 bootstrap replicates. *Edaphochlorella mirabilis* (LT560369) was used as an outgroup to root the tree. The sequence obtained from the studied Antarctic isolate, WCY_AQ5_1, was deposited in GenBank with accession number OQ225631.

#### 2.3.3. Characterisation of Cell Surface Properties

Cell surface properties of the studied microalgal strain were characterized using the microbial adherence to hydrocarbons (MATH) assay and Fourier transform infrared spectroscopy (FTIR). MATH analysis was used to study the physicochemical properties of the cell wall, such as the surface charge of the cell and degree of hydrophobicity. Chloroform is an acidic solvent, while ethyl acetate is a basic solvent. Comparison of cell adherence between these two solvents is commonly used to annotate the overall cell surface charges. Hydrophobicity is expressed as the percentage of adherence to hexadecane. MATH followed the method of Rosenberg et al. [27]. Cells were harvested by centrifugation and resuspended in 0.01 M potassium phosphate buffer (pH 6.8) with cell density ~0.4 at OD_600_ (*A*_0_). A volume of 0.4 mL of solvent (chloroform, ethyl acetate, and hexadecane) was added to 2.4 mL of cell suspension. The two-phase system was vortexed for 30 s and allowed to settle for 20 min. Absorbance of the aqueous phase was measured at 600 nm (*A*_1_). The percentage (*p*) of MATH was calculated as follows:

(5) $$p=(1-\frac{A_{1}}{A_{0}})\times 100$$

To provide a qualitative preliminary analysis of cell surface functional groups relevant for phytoremediation, FTIR was performed on algal culture samples before and after being treated with 1% *v*/*v* diesel, as described in Section 2.4. Cell pellets were collected and freeze dried for 48 h prior to FTIR analysis. Infrared absorption spectra scanned between 4000 and 400 cm^−1^ were recorded using FTIR (ALPHA, Bruker Optik GmbH, Ettlingen, Germany) through the attenuated total reflection (ATR) mechanism.

### 2.4. Phytoremediation of Diesel Hydrocarbons

Microalgal cultures were centrifuged at 8000× *g* and 4 °C for 15 min at the mid-logarithmic phase, and the cell pellets were then washed twice with 1 × PBS. The algal suspension was adjusted to an optical density of 0.5 at 620 nm (OD_620_) (1 × 10^7^ cells/mL). Fifty millilitres of fresh BBM were supplemented with 20% algal suspension and 1% *v*/*v* diesel (PETRONAS Dynamics Diesel Euro 5) filtered through a 0.22 µm membrane filter. The cultures were then grown for 9 d at 10 °C illuminated with cool white fluorescent lamps (42 µmol m^−2^ s^−2^ PAR) on a 12:12-h light–dark cycle. The diesel remediation achieved by the microalgal culture was examined at two-day intervals. An abiotic control was used to measure any abiotic loss of diesel.

To measure the phytoremediation efficiency, the total volume of the algal cultures (50 mL) was harvested and centrifuged at 8000× *g* for 15 min at 4 °C. The supernatant and algal pellet were extracted separately with *n*-hexane to quantitatively measure the residual diesel (W_f_) using gravimetric analysis, as described in Section 2.5.1. The amount of diesel extracted from supernatant reflects the percentage of residual diesel in the medium (A). The amount of oil extracted from the cell pellet represents the removal efficiency of adsorption (B). Calculations were made using the following equations:

(6) $$Percentage\;of\;residual\;oil\;(A)/Removal\;efficiency\left(B\right)\left(\%\right)=\frac{W_{f}}{W_{i}}\times 100$$

where W*_i_* = initial amount of oil and W*_f_* = amount of oil extracted from supernatant/pellet. The values of A and B were then used to calculate biodegradation efficiency (BE):

(7) $$BE\;\left(\%\right)=100-A-B-abiotic\;loss$$

### 2.5. Quantitative Assessment of Microalgal Biodegradation Efficiency

#### 2.5.1. Gravimetric Analysis

Gravimetric analysis was carried out following the incubation period via *n*-hexane extraction (1:1 medium to solvent ratio) of the cultures. The mixture was swirled and allowed to settle for 10 min. Then, the supernatant consisting of the solvent with residual oil was transferred into a pre-weighed glass dish (W_0_). The mixture was allowed to dry under a fume hood for 24 h before the final mass of the glass dish was measured (W_1_). The amount of oil extracted (W*_f_*) was calculated as: $W_{f}=W_{1}-W_{0}$. All samples were run in triplicate.

#### 2.5.2. Chromatographic Analysis

After measuring for the final mass, the residual extract was washed with 2 mL fresh *n*-hexane, filtered through Whatman paper No. 1, and stored in tared vials at 10 °C prior to gas chromatographic analysis. Quantitative and qualitative analyses of residual hydrocarbons in the extracts was conducted using a gas chromatograph (GC-2010 Plus, Shimadzu, Kyoto, Japan) equipped with an ZB–5MS capillary column (30 m × 0.25 mm × 0.25 μm) and a flame ionisation detector (FID). One microlitre of extract was injected into the GC–FID according to the modified specifications (Table 1).

The GC peaks for C_7_–C_30_ were manually integrated with the external *n*-alkane standards (Sigma-Aldrich, St. Louis, MO, USA) using Shimadzu LabSolutions CS software version 5.54 SP5. The concentration of each alkane in the sample was calculated based on the chromatogram peak area as compared to the external standards. The percentage of hydrocarbons mineralised relative to the abiotic loss of hydrocarbons in experimental controls was calculated using the equation:

(8) $$Mineralised\;hydrocarbons\left(\%\right)=\left(\frac{A_{ac}}{A_{i}}\times 100\right)-\left(\frac{A_{s}}{A_{i}}\times 100\right)$$

where $A_{ac}=$ total peak area in abiotic control, $A_{s}=$ total peak area in sample, $A_{i}$ = total peak area in 1% *v*/*v* diesel.

Gas chromatography-mass spectrometry (GC-MS) (QP-2010 Ultra, Shimadzu) was used to analyse other compound mass spectra based on the National Institute of Standards and Technology (NIST) mass spectral library.

### 2.6. Statistical Analyses

Each experiment was conducted in triplicate, and mean values and standard deviations (SD) were calculated. Statistical analysis was performed using GraphPad Prism version 8 (GraphPad Software Inc., Boston, MA, USA). One-way analysis of variance (ANOVA) was followed, where significant, by Tukey LSD multiple comparisons and the Wilcoxon test when non-parametric tests were necessary.

## 3. Results

### 3.1. Polyphasic Identification

**Figure 1:**
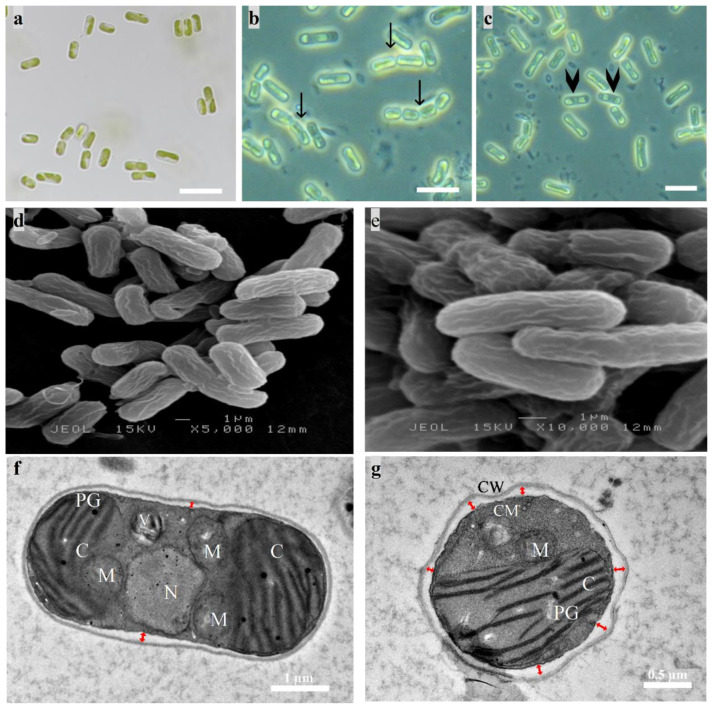
Micrograph images of raw algal isolate under normal cultivation conditions. (**a**) Light microscopy image at 1000× magnification; (**b**,**c**) phase contrast microscopy image. Black arrows indicate two to four solitary cells forming a short filament, and arrowheads indicate the presence of two chloroplasts located at the polar sides of one vegetative cell. Scale bar = 10 µm; (**d**,**e**) SEM micrographs showing the rough surface of the cylindrical cells with striated wall; (**f**,**g**) TEM micrographs of longitudinal and transverse sections of the cell. Red arrows indicate the periplasmic space. C, chloroplast; N, nucleus; PG, plastoglobuli; M, mitochondria; V, vacuole; CW, cell wall; CM, cell membrane.

**Figure 2:**
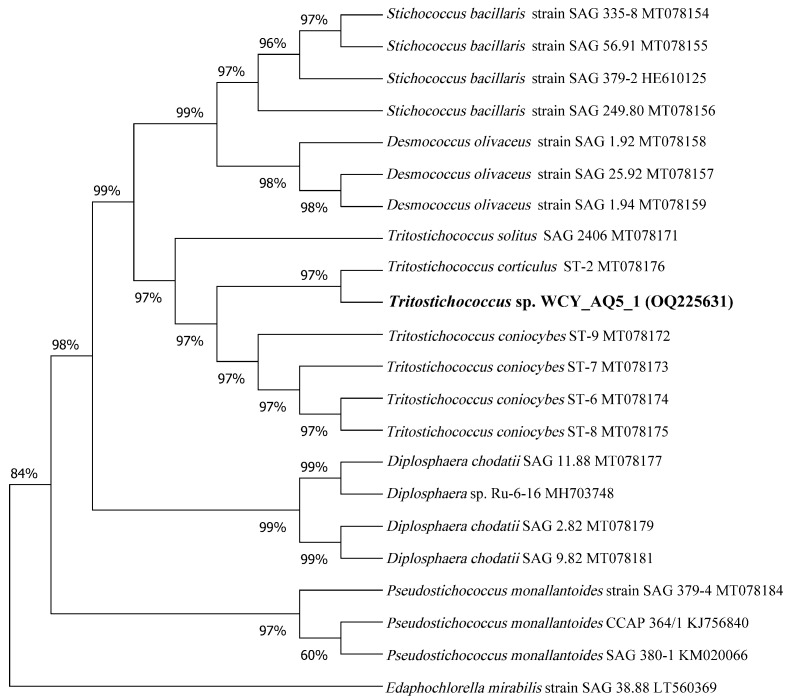
Molecular phylogeny of strain WCY_AQ5_1 (in boldface). The phylogenetic tree was inferred using the maximum likelihood approach and Kimura 2-parameter model. Numbers next to branches indicate bootstrap values. *Edaphochlorella mirabilis* was used as the outgroup.

The cell morphological features conformed closely to the genus *Tritostichococcus* [25]. Cells were cylindrical with rounded ends (Figure 1). Cells were unicellular or uniseriate with few cells. Short, loose, slightly curved filaments were composed of two to four cells (Figure 1b). Cells were (3) 4–12 (16) µm long and (1.3) 1.8–2.6 (3.0) µm wide with a length/width ratio of 2–4.6. The single parietal chloroplast occupied more than half of the cell with the absence of pyrenoid. Occasionally, two chloroplasts were situated at the polar parts of a single cell (Figure 1d,f). SEM images showed a rough cell surface with striated cell wall (Figure 1d). A thin layer of cell wall (20–30 nm) with low electron density was apparent in TEM with no mucilaginous sheath. The periplasmic space reached up to 0.14 µm given the irregular shape of the cell wall (Figure 1g). The morphological identification was supported by molecular analysis based on the concatenated 18S rDNA–ITS sequence. The length of the sequence obtained was 2758 bp. The ML tree (Figure 2) revealed that strain WCY_AQ5_1 (OQ225631) belonged to the well-supported *Prasiola* clade of the Trebouxiphyceae (Chlorophyta). The strain was located within the recently erected *Tritostichococcus* clade. Within this clade, the strain shared the same lineage with *T. corticulus*, supported by a high bootstrap value. The BLAST result also showed the highest percent identity (99.76%) and highest query cover between strain WCY_AQ5_1 and *T. corticulus* (MT078176). As both morphological and molecular identifications were consistent, strain WCY_AQ5_1 is confidently assigned to the genus *Tritostichococcus.*

### 3.2. Surface Property Characterisation

**Figure 3:**
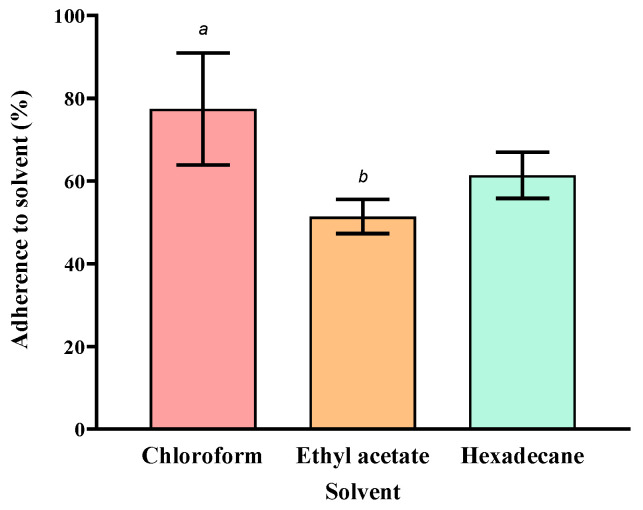
Results of MATH analysis using chloroform, ethyl acetate, and hexadecane. Mean ± SD (*n* = 3) values are indicated. Different letters indicate statistically significant differences.

MATH analysis was used to study the physicochemical properties of the cell wall. The alga showed higher affinity to chloroform than ethyl acetate, annotating the overall negatively charged surface (Figure 3). Cell surface hydrophobicity was determined through the partitioning of cells between water and hexadecane, an *n*-alkane solvent. The percentage of adhesion to hexadecane was 61.4%.

**Figure 4:**
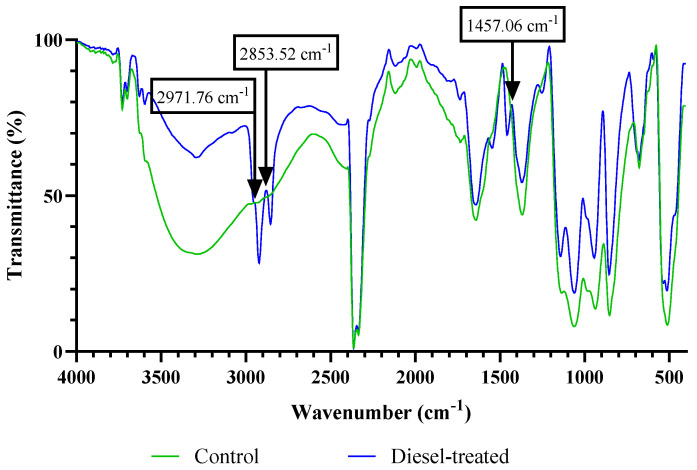
FTIR spectrum of isolate WCY_AQ5_1 before and after exposure to diesel. Arrows indicate the appearance of new peaks on the treated alga, suggesting the absorption of diesel alkane on to the cell surface.

Changes in the surface functional groups vibrations of raw and diesel-treated algae were reflected by the different vibrational frequencies observed in ATR-FTIR spectra (Figure 4). The different characteristic peaks indicated the possible presence of amino, carboxylic, hydroxyl, and carbonyl groups. The range of major FTIR band assignments for algal analysis is presented in Table 2. After exposure to diesel, new peaks were observed at 2971.76, 2853.52, 1457.06, and 1141.70 cm^−1^, which suggested the stimulation of surface lipid groups and proteins. As well as the band assignments to biomolecules, the appearance of new peaks at 2971.76, 2853.52, and 1457.06 cm^−1^ (indicated with arrows in Figure 4) also evidenced the adsorption of diesel onto the algal cell surface.

### 3.3. Efficiency of Phytoremediation of Diesel

#### 3.3.1. Total Hydrocarbon Loss

**Figure 5:**
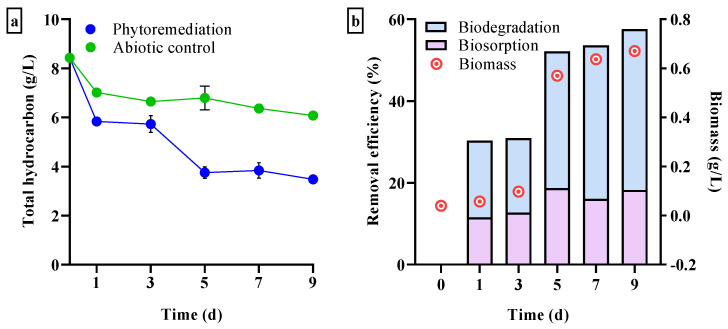
Temporal changes in total hydrocarbon loss due to phytoremediation and natural attenuation (abiotic control) during the nine-day incubation period. (**a**) Concentration of residual total hydrocarbon in the medium containing 1% *v*/*v* diesel; (**b**) cell growth based on dry biomass and phytoremediation efficiency due to biosorption and biodegradation. Mean ± SD (*n* = 3).

To assess the bioremediation ability of the studied microalgal strain against diesel, the algal culture was incubated with 1% *v*/*v* diesel for nine days and the gravimetric data were assessed at two-day intervals (Figure 5). A significant reduction in the abiotic control was only recorded on the first day of the experiment (~1.46 g/L), with no significant differences detected between any of the subsequent days (Figure 5a). The amount of diesel loss in phytoremediation over natural attenuation increased from 1.18 g/L on day 1 to 3.04 g/L on day 5, after which no further significant bioremediation was observed.

Phytoremediation includes a combination of biosorption and biodegradation. After the effects of abiotic losses (e.g., evaporation and attachment to flask walls) were eliminated, the removal of diesel initially took place at a slower rate during the first three days of incubation, with biosorption and biodegradation contributing ~12% and ~18%, respectively (Figure 5b). Biodegradation efficiency increased 1.8-fold to 33% (*p* < 0.05) on day 5. This was consistent with the exponential growth phase of cells based on dry biomass analysis. Final biodegradation efficiency was 39.3%. Biosorption remained in the range 11–18% throughout the incubation period and did not differ significantly. Total hydrocarbon content was reduced to 3.47 g/L, indicating that 57.6% of the initial diesel hydrocarbons were removed solely by phytoremediation over the nine-day period (with no further significant removal after five days).

#### 3.3.2. Residual Hydrocarbon Profile

**Figure 6:**
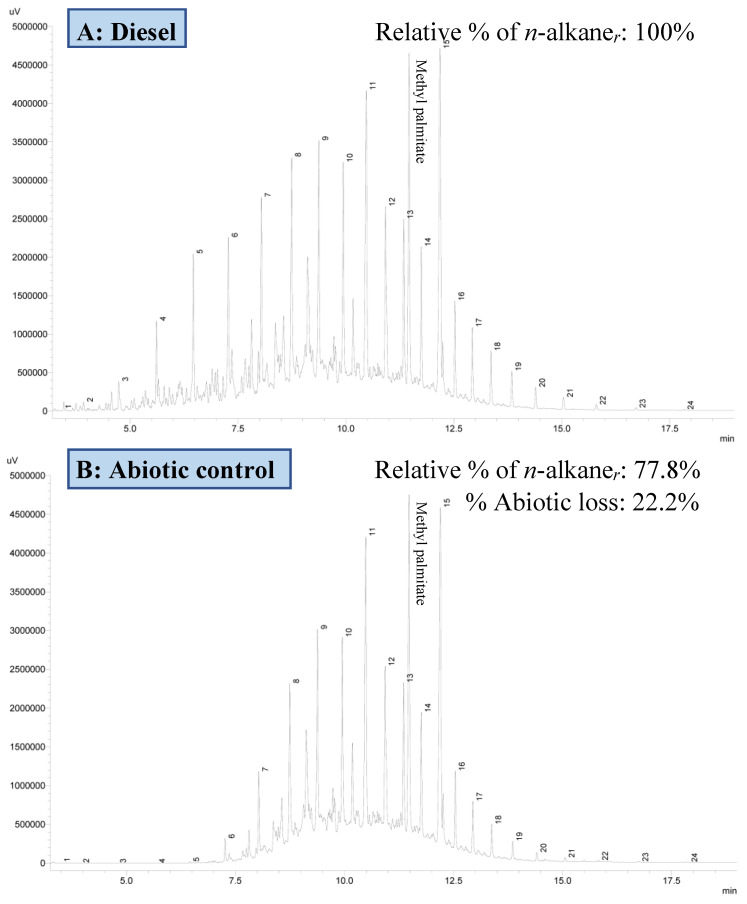

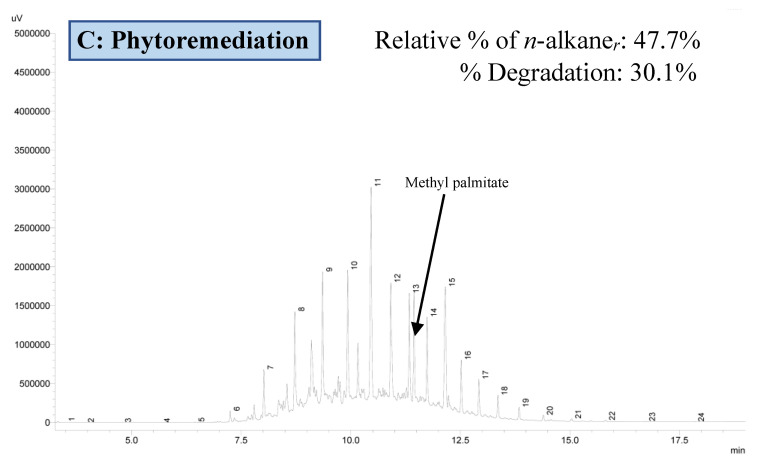
GC-FID chromatograms of (**A**) 1% (*v*/*v*) diesel; (**B**) abiotic control; (**C**) phytoremediation culture. Numbers above each peak represent the *n*-alkane, ranging from C_7_ to C_30_. Methyl palmitate (C_17_H_34_O_2_) was identified using GC-MS based on mass spectral library. *n*-alkane*_r_* = residual *n*-alkane in the medium.

The residual hydrocarbon profile at the end of the incubation was analysed using GC-FID (Figure 6). The residual hydrocarbon present in the abiotic control was 77.8%, indicating 22.2% abiotic loss, consistent with the gravimetric data. The residual hydrocarbon present in the phytoremediation study was 47.7%, meaning that 30.1% of the initial hydrocarbon was biologically degraded. This differed from the gravimetric estimate of biodegradation by 9%. Such differences could be attributed to the loss of other hydrocarbon derivatives in the degradation process, such as alkenes and aromatic hydrocarbons. One such example is the presence of an unintegrated peak near C_20_ (peak numbered 13), which GC-MS identified as hexadecanoic acid methyl ester (also known as methyl palmitate) based on the NIST mass spectral library. According to the Department of Standards Malaysia [30], Petronas Euro 5 diesel contains a B7 mix (7% methyl palmitate and 93% regular diesel), consistent with the presence of this compound on the chromatogram. A clear reduction in methyl palmitate was observed throughout the study suggesting its degradation, while this loss was not integrated into the calculation of relative percentage degradation. Overall, the chromatogram clearly shows that strain WCY_AQ5_1 could degrade a range of hydrocarbon compounds of short and mid-chain lengths. Notably, the microalga degraded 3483.66 ± 30.96 µg/mL of *n*-heneicosane (peak numbered 15), 57% of its initial concentration in the sample. No significant degradation of long chain alkanes (C_22_ to C_30_) was detected.

### 3.4. Physiological Effects on Microalgal Cells Exposed to Diesel

#### 3.4.1. Growth Response and Changes in Photosynthetic Pigment Content

**Figure 7:**
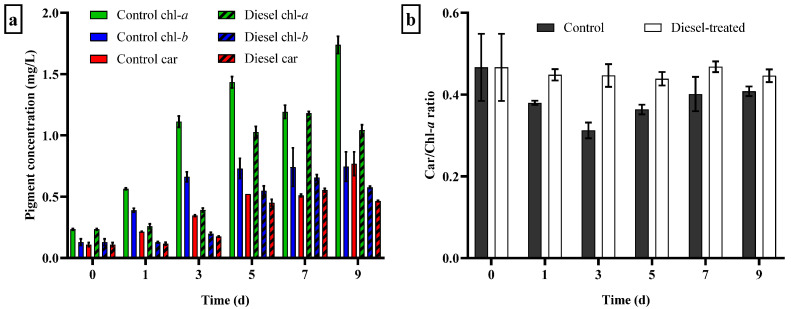
Temporal changes in (**a**) photosynthetic pigment contents (chlorophyll *a* (chl-*a*), chlorophyll *b* (chl-*b*), and total carotenoids (car)); (**b**) carotenoid-to-chlorophyll *a* ratio (car/chl-*a*) between control and diesel-exposed cells.

A clear correlation was found between algal dry mass and chl-*a* concentration in the diesel-treated cells (*R*^2^ = 0.97). Under control conditions, a lag phase was not observed, and the cells grew exponentially (*µ* = 0.34 ± 0.016 day^−1^). A lag phase was clearly apparent in the first three days in the diesel-treated culture, indicating the adaptation of cells in the presence of xenobiotics (Figure 7a). The specific growth rate was lower (*µ* = 0.253 ± 0.014 day^−1^), and the culture reached stationary phase more rapidly than the control. In general, the photosynthetic pigment contents of the microalgal cells cultivated in the presence of diesel were 1.2 to 2-fold lower compared to the control. Contents of all three pigments assessed (chl-*a*, chl-*b*, total carotenoids) showed similar sigmoidal curves over time. The difference in chl-*b* content was more pronounced as compared to the control. The total carotenoid content was not significantly different between diesel-exposed and control cultures.

The carotenoid-to-chlorophyll *a* ratio (car/chl-*a*) is commonly considered as a stress marker associated with oxidative damage. This ratio value was significantly different (*p* < 0.05) between the control and diesel-exposed cells (Figure 7b). The car/chl-*a* ratio in the control culture initially decreased to 0.30 and subsequently increased to 0.40 on day 9. The car/chl-*a* ratio of diesel-treated cells was slightly higher than that of the control, ranging from 0.43 to 0.48. This value remained consistent throughout the experiment.

#### 3.4.2. TEM Imaging of Cellular Response to Diesel Exposure

**Figure 8:**
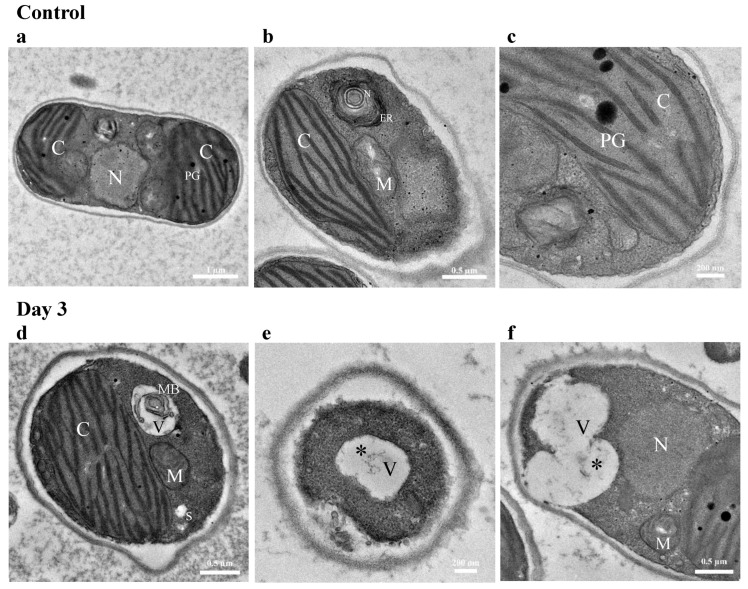

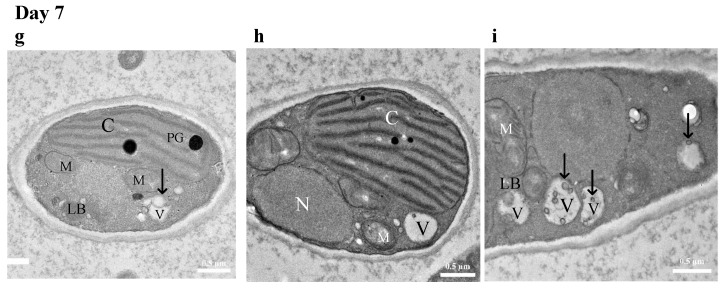
Transmission electron microscope (TEM) images of *Tritostichococcus* sp. WCY_AQ5_1 grown in (**a**–**c**) control condition, illustrating changes in ultrastructure on (**d**–**f**) day-3 and (**g**–**i**) day-7 after exposure to diesel. C, chloroplast; N, nucleus; PG, plastoglobuli; M, mitochondria; V, vacuole; MB, multilamellar body; S, starch granules; LB, lipid body. Asterisks (*) indicate sporadic granules in the vacuoles. Arrows indicate single membrane-bounded vesicles in the vacuoles.

Figure 8 shows a series of TEM micrographs of diesel-treated algal cells harvested on days 3 and 7 of the incubation period. These were compared with cells grown under control conditions (Figure 8a–c). Ultrastructural integrity of chloroplasts was retained with well-developed thylakoid membranes and few plastoglobuli in all samples. Lipid bodies and/or carbohydrate reserves were not discernible in cells from the control culture with small numbers being observed on day 7 in the diesel-exposed culture (Figure 8g,i). Reorganization of vacuoles was clear in microalgal cells in response to diesel exposure. Various inclusions of different sizes and morphologies were distinct against the relatively electron-transparent background of the vacuolar matrix (Figure 8d–i). Grain-like depositions were scattered over the vacuolar interior and around the inner side of the tonoplasts (Figure 8e,f). In addition to the presence of granular depositions, an increased number of small vacuoles containing spherules were present in proximity to the plasma membrane on day 7 (Figure 8g–i). Several single membrane-bound vesicles were located around the vacuoles. Some vacuoles showed modest autophagic activity (Figure 8f). Autophagic activity in microalgal cells is commonly associated with lipid droplet formation. A multilamellar body was also present within the vacuole (Figure 8d).

## 4. Discussion

*Stichococcus*-like microalgae are characterised by a simple rod-shaped morphology and are widespread in terrestrial and freshwater habitats [26]. They show high phenotypic plasticity in cultivation under different conditions, which has led to considerable taxonomic confusion since their first description by Nägeli (1849) [31]. Most recently, Pröschold and Darienko (2020) [25] redefined the genus *Stichococcus*, erecting eight independent genera within the *Prasiola* clade (Treubouxiophyceae). In this study, the Antarctic algal strain, WCY_AQ5_1, was confidently assigned to the genus *Tritostichococcus*, one of the newly erected lineages.

Microalgae now assigned to *Tritostichococcus* have been previously reported from North America, South America, and Europe [25] but only recently from Antarctica [32]. Based on the available molecular data, strain WCY_AQ5_1 is closely related to *T. corticulus;* however, it differs from the morphological description of *T. corticulus*, which contains a pyrenoid [25]. The latter was also described to be single-celled with a smaller length/width ratio (1.7–2.9). Molecular information is currently limited by the partial sequence obtained in this study; hence, the strain could only be discriminated at the generic level. Further ITS-2 secondary structure analysis is required to further distinguish taxa within the genus. Although biological data on algae now assigned to *Tritostichococcus* are scarce, members of *Stichococcus* and other closely related genera are known to be highly tolerant to desiccation and osmotic stress [33]. A recent study of several strains of *Tritostichococcus*, including from the South Orkney Islands in maritime Antarctica, reported strong resistance towards the antibiotic cycloheximide [32].

Microalgae have been applied in wastewater decontamination through heavy metal removal [34,35,36]. The strong metal-binding property that is characteristic of many microalgae is associated with their generally negatively charged surfaces, as also exhibited by the microalga studied here. Using the MATH protocol, the degree of cell surface hydrophobicity can be compared with other algae. In other studies, members of the genera, *Chlorella, Desmodesmus, Monoraphidium,* and *Microcystis*, were reported to show 5–50% adhesion to *n*-alkane solvent [37,38]. Relative to these studies, the adhesion of strain WCY_AQ5_1 to hexadecane was somewhat higher (~61%), demonstrating a strongly hydrophobic property. High hydrophobicity has been correlated to the rate of biodegradation achieved in some microbial bioremediation studies [39,40]. FTIR results corroborated the cell adsorption capability with the appearance of new peaks upon diesel exposure. Absorption peaks of diesel alkanes were previously reported at 2922.03, 2852.59, and 1459.09 cm^−1^, corresponding to C-H stretching and C-H bending [41]. These results confirm the mechanism of adsorption, which is dependent on cell surface properties.

Biosorption, bioaccumulation, and biodegradation are three common elements of phytoremediation with their kinetics differing between microalgae [15]. For instance, in an evaluation of the removal of benzo(a)pyrene, *Selenastrum capricornutum* predominantly degraded the hydrocarbons and rapidly formed metabolites, whereas *Scenedesmus acutus* had higher sorption capability over degradation [42]. In the current study, strain WCY_AQ5_1 demonstrated biosorption as a facilitating process for biodegradation in the removal of diesel hydrocarbons. Notably adsorption, which commonly increases with cell abundance, did not follow this pattern, and increasing cell biomass from day 3 onwards did not significantly enhance diesel biosorption. This observation may be underlain by distinct growth characteristics of microalgal cells in different cultivation modes. Variations in cell size have been reported in photoautotrophic, mixotrophic, and heterotrophic conditions due to physiological acclimation associated with energy acquisition [43,44]. We therefore speculate that larger microalgal cell size, concomitant with smaller surface-area-to-volume ratio may minimise differences in the total amount of oil adsorbed onto the total cell surface available in the cultures during the exponential phase.

The *n*-alkane degradation profile of strain WCY_AQ5_1 was similar to those of *Prototheca zopfii* and *C. vulgaris*, which were able to degrade alkanes from C_12_ to C_21_ [45,46]. Diesel fuel contains a complex mixture of hydrocarbons, although the majority are C_10_ to C_23_. In our study, the proportion of certain residual hydrocarbons was sometimes greater than the control level, which may indicate the ability of the microalga to convert some components to intermediate compounds, forming a dynamic hydrocarbon pool [47,48]. The degradation of aromatic compounds was not a focus of this study. Nevertheless, various previous studies have confirmed the degradation of aromatic compounds, such as pyrene and fluoranthene, using a variety of microalgal strains [17,42,49].

Reduction in photosynthetic pigment content when exposed to diesel is a phenomenon previously described during the transition from phototrophic to mixotrophic growth conditions [50]. Strain WCY_AQ5_1 was capable of using diesel as a metabolic carbon source under a 12:12 h light–dark mixotrophic regime. This is consistent with the study of Cheirslip and Torpee (2012) [51] who reported that photosynthetic pigment concentrations were influenced by both the availability of a suitable organic carbon source and the illumination regime to which the cells were subjected. They showed that algal biomass and lipid production were enhanced, while chlorophyll content was lower in mixotrophic growth than in photoautotrophic culture but higher than in heterotrophic culture.

Changes in pigment composition were also observed in this study. The rates of primary and secondary pigment production were similar, as suggested by the consistent car/chl-*a* ratio throughout the diesel incubation period. The initial drop in ratio value can be explained by the rapid production of chl-*a* as the main photosynthetic pigment in green microalgae signifying active growth, whereas the increased carotenoid production as the culture aged was likely necessary to enhance light harvesting and combat reactive oxygen species. The higher carotenoid productivity compared to the control was likely to be a photoprotective strategy as observed in stressed cells. Biosynthesis of carotenoids could be stimulated by the formation of lipid bodies in the cell as a protective role against peroxidation of unsaturated lipids [52]. Cytoplasmic lipid bodies also provide storage for the accumulation of β-carotene [53]. Although there was no microscopically visible accumulation of lipid droplets in the studied algae, TEM imaging showed progressive vacuolar activities that indicated production of lipids. Other studies have also reported the transformation of the multilamellar body from organelles as an acclimation response in nutrient-starved or heavy-metal-stressed cells [54,55]. The multilamellar body would lose its structure in the autophagic vacuoles and eventually fuse into a single lipid mass. These granular inclusions are typically found in many microalgae and play a key role in the localisation of polyphosphate [56]. Polyphosphate metabolism is integral to various cellular processes, including acclimation of cells to environmental and biotic stress [57]. Mixotrophic and heterotrophic cultivation of microalgae results in greater production of lipids, especially those composed of high-quality polyunsaturated fatty acids that are valuable in biodiesel production [58,59,60].

## 5. Conclusions

The present study confirms that the Antarctic microalgal strain, *Tritostichococcus* WCY_AQ5_1 (OQ225631), is capable of remediating diesel hydrocarbons, potentially stimulating lipid production during the process. The cultivation of microalgae using wastewater effluent to concurrently remove pollutants and enhance algal biomass and biocompound production is a relatively new biotechnological process but is showing promising results. The integration of pollution management and biocompound production through microalgal research could be environmentally sustainable at the same time as unlocking considerable economic rewards.

## Figures and Tables

**Table 1:** Specifications of GC-FID for chromatographic analysis of diesel hydrocarbons and external standard C_7_–C_30_ (adapted from [9]).

Specifications	
Oven program	Initial: 40 °C Ramp: 20 °C/min until 200 °C then 30 °C/min until 300 °C (10 min hold time)
Injection mode	Split (10:1 ratio)
Carrier gas	Helium
Gas flow rate	Helium: 30 mL/min Hydrogen: 40 mL/min Compressed air: 400 mL/min
Column flow rate	0.9 mL/min
Pressure	73.9 kPa
Back detector (FID)	310 °C

**Table 2:** Assignment of FTIR spectra of strain WCY_AQ5_1 before (control) and after diesel exposure [28,29].

Main Peaks (cm^−1^)		Tentative Band Assignment	Biomolecule	Wavenumber Range (cm^−1^)
Control	Diesel-Treated			
**3276.99**	3294.07	ν O-H/ν N-H	Water, protein	3639–3029
**-**	2971.76, 2853.52	ν_as_ CH_2_, ν CH_2_, ν CH_3_	Lipid, proteins	2970–2850
**1640.04**	1639.95	ν C=O of amide I	Proteins	1655–1638
**-**	1457.06	δ_as_CH_3_, δ_as_CH_2_	Lipid, proteins	1456–1450
**1368.67**	1370.58	Δ CH_3_	Lipid, proteins	1398–1370
**1061.31**	1141.70, 1060.49	P=O	Phospholipids, nucleic acids	1200–900
**936.75, 853.09**	943.12, 854.69	ν (C-O-C) of polysaccharides	Nucleotides, carbohydrates	

ν = symmetric stretch, ν_as_ = asymmetric stretch, δ = symmetric deformation (bend), δ_as_ = asymmetric deformation (bend).

## Data Availability

Not applicable.
